# The Emerging Use of ASC/Scaffold Composites for the Regeneration of Osteochondral Defects

**DOI:** 10.3389/fbioe.2022.893992

**Published:** 2022-06-30

**Authors:** Gohar Rahman, Trivia P. Frazier, Jeffrey M. Gimble, Omair A. Mohiuddin

**Affiliations:** ^1^ Dr. Panjwani Center for Molecular Medicine and Drug Research, International Center for Chemical and Biological Sciences, University of Karachi, Karachi, Pakistan; ^2^ Obatala Sciences Inc., New Orleans, LA, United States

**Keywords:** adipose-derived stem cells, osteochondral defects, stem cells, scaffold, tissue engineering

## Abstract

Articular cartilage is composed of chondrocytes surrounded by a porous permeable extracellular matrix. It has a limited spontaneous healing capability post-injury which, if left untreated, can result in severe osteochondral disease. Currently, osteochondral (OC) defects are treated by bone marrow stimulation, artificial joint replacement, or transplantation of bone, cartilage, and periosteum, while autologous osteochondral transplantation is also an option; it carries the risk of donor site damage and is limited only to the treatment of small defects. Allografts may be used for larger defects; however, they have the potential to elicit an immune response. A possible alternative solution to treat osteochondral diseases involves the use of stromal/stem cells. Human adipose-derived stromal/stem cells (ASCs) can differentiate into cartilage and bone cells. The ASC can be combined with both natural and synthetic scaffolds to support cell delivery, growth, proliferation, migration, and differentiation. Combinations of both types of scaffolds along with ASCs and/or growth factors have shown promising results for the treatment of OC defects based on *in vitro* and *in vivo* experiments. Indeed, these findings have translated to several active clinical trials testing the use of ASC-scaffold composites on human subjects. The current review critically examines the literature describing ASC-scaffold composites as a potential alternative to conventional therapies for OC tissue regeneration.

## 1 Introduction

Tissue engineering is an interdisciplinary field which amalgamates the applications and principles of life sciences and engineering to develop biological substitutes to maintain, improve, or restore tissue function (Langer and Vacanti, 2016). While the body generally has good self-healing potential, the extent of self-repair varies among different tissues and may also be affected by diseases or injuries (Lanza et al., 2020). Tissue engineering involves the use of cells, scaffolds, and/or bioactive molecules to integrate and perform tissue repair (Lee et al., 2014). A substantial challenge associated with the implantation of cells alone into the body is the uncertainty of cellular fate post-implantation. Unlike drugs whose actions can be correlated with the physiological response, for stem cells, there is a need to track, quantify, and check the viability of the cells at the desired site of action (Nguyen et al., 2016). Moreover, there is a loss of implanted cells from the site of action due to systemic resorption and damage by the inflammatory microenvironment (Afessa and Peters, 2006). Therefore, to enhance the retention and viability of cells at the site of tissue injury, scaffolds and biological factors serve as useful adjuvants to cell therapy alone. Scaffolds can be broadly divided into two categories, either natural or synthetic. Regardless of their origin, scaffolds are intended to support the cell’s attachment, proliferation, migration, and differentiation (Keane and Badylak, 2014). Currently, there are multiple clinically relevant biomaterials for tissue engineering applications of skin, cartilage, bone, and heart available in the market (Lee et al., 2014).

The present article is intended to provide an overview of the recent literature focused on the application of adipose-derived stromal/stem cells (ASCs) and scaffold composites for the treatment of OC defects. The search terms used to review the primary literature were “adipose-derived stem cells,” “osteochondral defects,” “scaffold,” and “hydrogel.” Studies were included if the ASC-seeded scaffolds were evaluated in the context of OC defect regeneration; in contrast, those studies conducted only on bone or cartilage defects were excluded.

## 2 Osteochondral Defects

The damage to the articular cartilage and the underlying subchondral bone leads to defects known as OC defects. These defects can result from aging, physical trauma, or chronic diseases such as osteochondritis or osteoarthritis (OA) (Hunter et al., 2014). While investigators initially believed that OA only adversely affected the articular cartilage (AC), it is now established that OA causes damage to all tissues within the diarthrodial joint, including ligaments, joint capsule, menisci, subchondral bone, and synovial membrane (Torres et al., 2006; Krasnokutsky et al., 2011). The impaired crosstalk between the AC and subchondral bone is a complex phenomenon, capable of inducing adverse biochemical and biomechanical changes in the osteochondral region (Hu et al., 2020). Such changes can result in the development of diseases including osteosclerosis, osteonecrosis, and osteochondritis in the subchondral regions. The histological changes that appear in the subchondral bone are a consequence of impaired bone mineralization and turnover, thus reducing overall bone density and subchondral bone volume. Moreover, these effects lead to alteration in the biomechanical properties of the osteochondral unit, thereby reducing the load-bearing capability of the osteochondral unit. The exact cause of OC defects is yet to be determined; however, it is generally believed that abnormal endochondral ossification, genetic factors, and repetitive microtrauma play a significant role in disease progression (Grimm et al., 2014). Furthermore, some studies have suggested that either repetitive stress on the osteochondral unit or a single acute event can trigger AC degradation *via* the expression of proteolytic enzymes. Induced expression of disintegrin and metalloproteinase with thrombospondin motifs (ADAMTS) and matrix metalloproteinase (MMP) family proteins can degrade the matrix composition, thereby contributing to the disease pathogenesis (Buckwalter et al., 2013; Chang et al., 2019).

The AC is a porous, avascular, and aneural structural organization composed of chondrocytes surrounded by an extracellular matrix (ECM) containing proteoglycans and collagen type II (Clouet et al., 2009). The organization and composition of the ECM control the mechanical properties of the AC (Sanchez-Adams et al., 2014; Prein et al., 2016). Because of the avascular nature of the AC, it lacks the ability to heal spontaneously when injured. In the United States and Europe, two million people are diagnosed with AC defects annually (Mumme et al., 2016). Nevertheless, there are no effective treatments available for AC injuries (Boyer et al., 2020).

**FIGURE 1:**
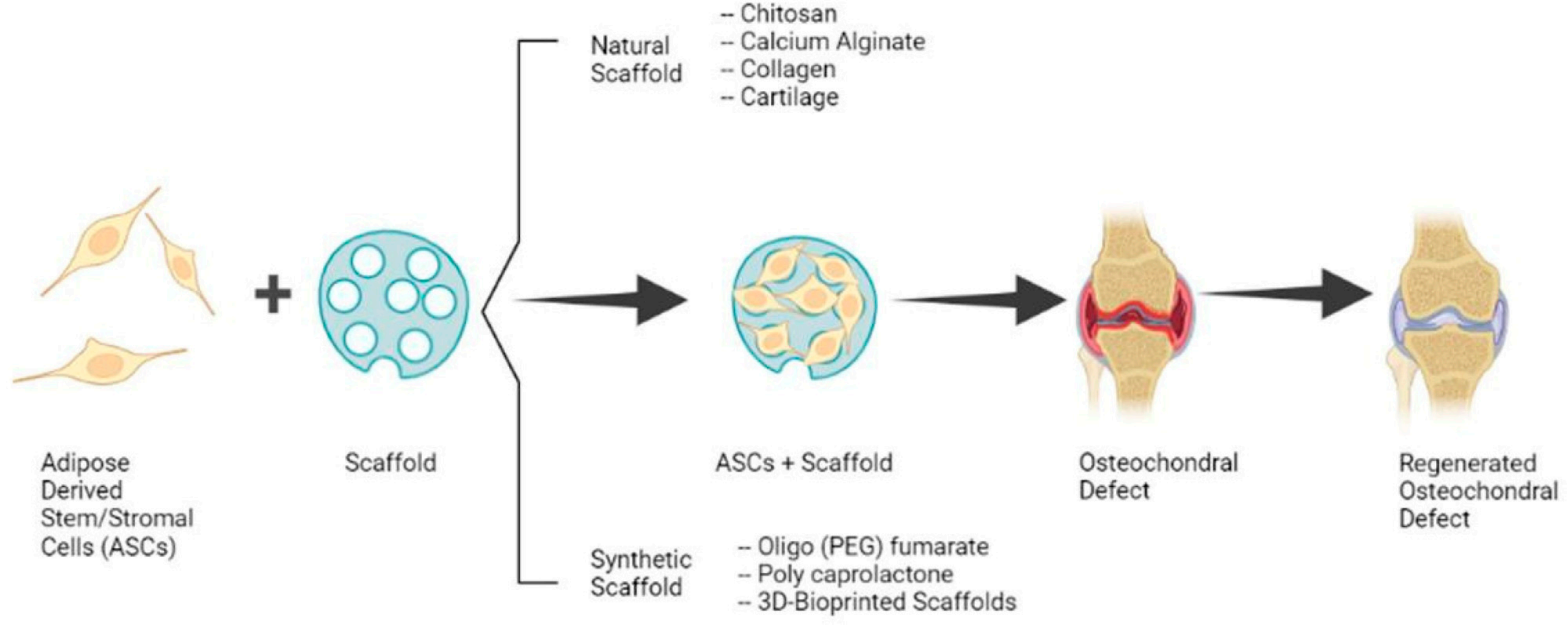
Clinical application of ASCs and Scaffolds for the Regeneration of OC Defects. (Image created with Biorender.com).

The present standard of care for OC defects includes microfracture which often results in the formation of fibrocartilage and is unsuitable for the treatment of large defects (more than 2–4 cm^2^). Possible therapy for large defects is the OC autograft transfer system which results in better healing; however, this procedure is technically difficult, has a risk of donor site morbidity, and the transplanted cartilage may fail to integrate. While autologous chondrocyte implantation has shown satisfactory results in the repair of hyaline-like cartilage, it is expensive and laborious because of the need for chondrocyte isolation and expansion *in vitro* (Chimutengwende-Gordon et al., 2020). Because of the limitations of these established therapies, recent studies have concentrated on the clinical translation of ASCs and scaffolds alone or as composites for the regeneration of OC defects (Figure 1).

## 3 Adipose-Derived Stromal/Stem Cells

Adipose tissue is present in humans diffusely throughout the body with major depots located in the abdomen, buttocks, thighs, arms, and breasts within both subcutaneous and intraperitoneal compartments. The main function associated with adipose tissue historically has been to store excess energy in the form of triglycerides within adipocytes. There has been an increased appreciation of adipose tissue as an endocrine organ since it secretes adipokines which modulate appetite and insulin sensitivity (Rosen and Spiegelman, 2014). In addition, adipose tissue regulates the body temperature and glucose and lipid homeostasis (Seale et al., 2011; Kajimura et al., 2015). There are two main types of adipose tissues, the white and beige/brown adipose tissue; however, in tissue engineering and regenerative medicine, the white adipose tissue is used more frequently because of its greater availability in the adult human population (Minteer et al., 2012). The ASCs isolated from white adipose tissue have the potential to differentiate into a variety of cell types including adipocytes, osteoblasts, chondrocytes, cardiac myocytes, and skeletal myocytes (Zuk et al., 2001; Lin et al., 2006; Estes et al., 2010; Boyer et al., 2020). In addition, the ASCs have been extensively investigated as adult stromal/stem cells for cartilage (Estes et al., 2010) and bone tissue-engineering applications (Rada et al., 2011). In addition to their differentiation capability, ASCs have a low immunological reactivity due to the low expression or absence of immunogenic surface antigens including cluster of differentiation (CD) 40, CD40L, CD80, CD86, and major histocompatibility complex (MHC) II (Bourin et al., 2013). The ASCs retain their low immunological reactivity even after osteogenic differentiation (Liu et al., 2013).

### 3.1 Source of Adipose-Derived Stromal/Stem Cells

Subcutaneous adipose tissue of arms, thighs, and abdomen are the most clinically relevant sources of ASCs (Bacakova et al., 2018; Louwen et al., 2018). The proliferation and differentiation of ASCs isolated from different physiological depots can vary (Fraser et al., 2007). For example, ASCs isolated from the medial thigh, trochanteric, or superficial abdominal regions and upper thigh show higher apoptosis than ASCs isolated from superficial abdominal regions (Schipper et al., 2008). It has been also reported that the isolation procedures can impact the ASC’s plasticity, functionality, and quality (Argentati et al., 2018; Palumbo et al., 2018). The main function of white adipose tissue is the storage of excess energy in the form of triglycerides and is found in visceral and subcutaneous adipose tissues (Himms-Hagen et al., 2000; Barbatelli et al., 2010). In terms of yield, the highest number of ASCs is obtained from arm adipose tissue while the highest plasticity is found in the white inguinal adipose tissue. In adults, brown adipose tissue is less abundant than white adipose tissues; however, brown adipose tissues can be found in neonates in the area of the neck, mediastinum, and interscapular tissues. In contrast to white adipose tissues, the primary function of beige/brown adipose tissues is thermogenesis or non-shivering heat generation. Consequently, ASCs derived from beige/brown adipose tissue display distinct characteristics compared with ASCs derived from white adipose tissue, including the ability to undergo skeletal myogenic differentiation (Raposio and Bertozzi, 2017).

### 3.2 Differentiation Capability of Adipose-Derived Stromal/Stem Cells

In tissue engineering and regenerative medicine, ASCs have significant utility because of their capability to differentiate along multiple lineages. The ASCs can efficiently differentiate into mesenchymal lineages such as bone, fat, and cartilage when exposed *in vitro* to appropriate inductive culture conditions. This is consistent with their ability to regenerate bone, fat, and cartilage *in vivo*. While there have been reports of ASC differentiation along non-mesodermal lineages (neurons, cardiomyocytes, and hepatocytes), the efficiency of non-mesodermal lineage ASC differentiation *in vivo* remains less well documented (Bunnell, 2021).

Compared with bone marrow-derived mesenchymal stem cells (BMSC), ASCs are considered inferior in the case of osteogenic differentiation *in vitro* (Brennan et al., 2017). However, it is well-established that ASCs undergo osteogenic differentiation both in vitro (Girolamo et al., 2007; Kuterbekov et al., 2018) and *in vivo* (Supronowicz et al., 2011; Schubert et al., 2013; Lo et al., 2017). Moreover, the differentiation of ASCs into bone and cartilage has also exhibited promising results in clinical applications (Sándor, 2012; Vériter et al., 2015; Farré-Guasch et al., 2018). It has been observed that the OC differentiation of ASCs can be enhanced by exogenous biological factors. The addition of growth factors such as transforming growth factor β1 (TGF-β1), TGF-β3 (Fan et al., 2010), and bone morphogenic protein 4 (BMP4) (Lópiz-Morales et al., 2010) enhance the OC differentiation of ASCs. It has also been shown in pre-clinical studies that the activation of fibroblast growth factor (FGF2) signaling enhances MSC-mediated cartilage repair (Grafe et al., 2018). Furthermore, additional studies have identified a correlation between cell culture oxygen levels and the differentiation and proliferation of ASCs. Generally, native chondrocytes and cartilage are cultured in low oxygen conditions ranging from 2–7% saturation, comparable with oxygen levels experienced by cartilage *in vivo* (Zhou et al., 2004). Consistent with these observations, ASCs display enhanced chondrogenesis when cultured *in vitro* under 5% oxygen (Merceron et al., 2010; Weijers et al., 2011).

## 4 Scaffolds in Tissue Engineering

In the field of tissue engineering, scaffolds serve as a fundamental component by virtue of their biochemical and mechanical properties. The scaffold composition and morphology influence cell adhesion, migration, proliferation, and differentiation (Litowczenko et al., 2021). Ideal scaffolds should mimic the natural environment, such that their pore size, surface area, porosity, and mechanical properties closely resemble those of the target tissue (Silva et al., 2007; Nava et al., 2016). In addition, scaffolds should be degradable and biocompatible (Gomes and Reis, 2004) with minimal risk of cytotoxicity or genotoxicity (Cvetković et al., 2018). Furthermore, ideal scaffolds should support attachment and proliferation by a wide range of cell types. It is desirable that the scaffolds are suitable for advanced functions such as encapsulation and release of bioactive factors, for example, growth factors, anti-inflammatory agents, and anti-bacterial molecules. Newly developed scaffolds with these versatile characteristics are showing promise in a variety of tissue engineering applications (Litowczenko et al., 2021) including the regeneration of the heart (Fang et al., 2020), skin (Khan et al., 2018; Maged et al., 2019), nervous tissues (Kuzmenko et al., 2018; Sadeghi et al., 2019), and bone tissue (Mohiuddin et al., 2019a; Zhang et al., 2019).

### 4.1 Scaffolds for Osteochondral Defect Regeneration

An optimal scaffold for OC regeneration should have the ability to support osteogenesis, chondrogenesis, and angiogenesis, and have space for nutrient and cell infiltration (Roddy et al., 2018). Scaffolds based on polymers, ceramics, and decellularized ECM have shown promising results so far for the regeneration of OC defects (Ghassemi et al., 2018; Roddy et al., 2018). The shape, size, total porosity, and pore interconnectivity significantly influence the therapeutic effect of a scaffold (Yamane et al., 2007). The pore structure is a critical feature among these properties since it is the literal “gatekeeper” of cell migration, growth, and nutrient flow (Zeltinger et al., 2001). Pore sizes that are too small will restrict cell migration, nutrient diffusion and waste removal and adversely affect cell viability, whereas pore sizes that are too large can diminish cell attachment and biomechanical stiffness of the scaffold (Karageorgiou and Kaplan, 2005). Presently, the two types of scaffolds used in bone and cartilage tissue engineering are synthetic and natural scaffolds.

#### 4.1.1 Synthetic Scaffolds

The most widely used synthetic scaffolds for OC regeneration include polylactide (PLA), polyglycolic acid (PGA), polylactic-co-glycolic acid (PLGA), poly-L-lactide (PLLA), polyethylene glycol (PEG), polyvinyl alcohol (PVA), and polycaprolactone (PCL). They are prepared as hydrogels or nanofibrous scaffolds (Sánchez-Téllez et al., 2017; Yang et al., 2017; Dai et al., 2018; Kudva et al., 2018; Critchley et al., 2020) which can be optimized for load-bearing tissues by adjusting their mechanical strength through varying concentrations and processing methods (Grigore, 2017). The stiffness of synthetic scaffolds is not only necessary for maintaining structural integrity and load-bearing *in vivo*, but it also provides physical cues for stem cells to differentiate along the OC lineage (Davis et al., 2021). One of the limitations of synthetic scaffolds is that they have a relatively low affinity for cell attachment compared to natural scaffolds. Therefore, to overcome this problem, different types of bioactive materials such as growth factors and/or peptides are added to improve cell attachment (Woodard and Grunlan, 2018). Among synthetic scaffolds, PEG is widely used because of its biocompatibility, hydrophilicity, inertness, and relatively low immunogenicity. Moreover, PEG has been shown to support the viability of chondrocytes along with the deposition of new ECM by the cells (Bryant and Anseth, 2002). PCL is an FDA-approved synthetic scaffold, which is widely used in TE because of its tunable mechanical strength. PCL can be prepared as an electrospun nanofibrous scaffold or porous scaffold depending on the desired application (Ding et al., 2014; Panadero et al., 2016). Ceramic scaffolds including tricalcium phosphate and hydroxyapatite are biocompatible and can promote osteoinduction but they are poorly absorbed and brittle (Ghassemi et al., 2018; Roddy et al., 2018). Synthetic polymers such as PLA are resorbable but have limited osteo-inductive capacity (Roddy et al., 2018).

#### 4.1.2 Natural Scaffolds

Scaffolds derived from natural sources used in chondral repair are generally prepared from collagen, hyaluronic acid, chitosan, silk, or alginate. The bioactivity, degradability, and biocompatibility of natural polymers make them desirable materials for TE (Jeuken et al., 2016; Li et al., 2018). Natural scaffolds are often prepared in the form of highly hydrated viscoelastic matrices known as hydrogels, which possess tunable swelling and mechanical properties based on the degree and type of cross-linking. These materials inherently provide the binding sites for cells, thus allowing cell-ECM interactions similar to native tissues (Catoira et al., 2019; Mantha et al., 2019).

Chitosan and alginate are among the natural polysaccharides that are widely used in cartilage repair and have displayed promising results (Xu et al., 2008; Yao et al., 2016; Ewa-Choy et al., 2017; Henrionnet et al., 2017; Merlin Rajesh Lal et al., 2017; Ruvinov et al., 2018; Huang et al., 2019). Based on our literature survey, chitosan has mainly been analyzed in combination for osteochondral defects and not alone. Alginate is derived from seaweeds, is both biocompatible and biodegradable, and is composed of beta-1-glucuronic acid and alpha-D-mannuronic acid. Several studies have reported that it supports chondrogenic proliferation, morphology, and the synthesis of type II collagen and proteoglycans (Homicz et al., 2003; Caron et al., 2012; Angelozzi et al., 2017; Aurich et al., 2018). Furthermore, stromal/stem cells derived from adipose, bone marrow, Wharton’s jelly, and dental pulp undergo chondrogenic differentiation when seeded on alginate scaffolds (Huang et al., 2015; Reppel et al., 2015; Ewa-Choy et al., 2017; Mata et al., 2017; Baba et al., 2018). The incorporation of mammalian compounds such as collagen into alginate has been shown to enhance the attachment and proliferation of cells (Bian et al., 2011; Lee and Mooney, 2012; Ganesh et al., 2013). Chitosan, prepared by the deacetylation of chitin, is another natural biomaterial used for OC regeneration. Because of its *in vivo* biocompatibility, degradability, and anti-bacterial properties, chitosan is widely used in TE (Cheung et al., 2015; Varun et al., 2017; Huang et al., 2019). It supports the chondrogenic differentiation of MSCs, chondrocytes, proliferation, and cartilaginous ECM deposition under *in vitro* and *in vivo* conditions (Griffon et al., 2006; Elder et al., 2013; Faikrua et al., 2013; Sheehy et al., 2015; Huang et al., 2019; Scalzone et al., 2019). However, chitosan presents poor mechanical properties requiring combination with other stiffer materials or the addition of crosslinking agents to optimize its activity in OC tissue engineering (Muzzarelli et al., 2015; De Mori et al., 2019; Kusmono and Abdurrahim, 2019; Scalzone et al., 2019; Islam et al., 2020; Jiang et al., 2022; Liu et al., 2022).

Gelatin methacryloyl (GelMa) hydrogels (semi-synthetic scaffold) have been widely investigated in TE because of their tunable physical and biological properties. The presence of cell attachment sites and MMP responsiveness allows stromal/stem cells to migrate and proliferate within GelMa in a manner similar to the native ECM (Yue et al., 2015). GelMa has been used in the regeneration of cardiovascular-like tissues (Chen et al., 2013), bone (Heo et al., 2014; Qiao et al., 2020), liver (Wu et al., 2020), and skin (Zhao et al., 2016).

Collagen is the most abundant ECM protein present in the animal kingdom. It provides high biocompatibility for cells and displays load-bearing capability due to its fibrous structure (Meyer, 2019). Consequently, collagen is used in many biological and medical products such as dental repair and wound healing (Costa et al., 2017). Nevertheless, collagen displays limitations in its physical properties such a lower mechanical strength than synthetic materials and its susceptibility to enzymatic degradation. Collagen modification by crosslinking to enhance stiffness and reduce degradability partially addresses these limitations and enhances its application for TE (Liu et al., 2019).

## 5 Adipose-Derived Stromal/Stem Cell-Scaffold Composites for Osteochondral Defect Regeneration

The ASCs’ mesenchymal differentiation capability is supported by a substantial body of literature demonstrating the efficacy and potential utility of ASCs for OC regeneration and defect repair (Bunnell, 2021). Since the direct application of ASCs to bone is hampered because of the stiffness and dry nature of the tissue, ASCs seeded on a scaffold presents a more practical mode of cell transplantation. Numerous biomaterials have been analyzed for their compatibility with ASCs and their subsequent capability to regenerate subchondral bone as composite biomaterials (Im et al., 2012; Lim et al., 2013; Boyer et al., 2020). Because of the favorable properties of hydrogels (biocompatibility, permeability, high water content, and tunable mechanical properties), they have been the most frequently studied biomaterial for OC defect regeneration (Guan et al., 2017).

### 5.1 Adipose-Derived Stromal/Stem Cell–Chitosan Scaffold

Chitosan has the ability to support cartilaginous ECM deposition and chondrocyte attachment which makes it a favorable scaffold for OC regeneration. The *in vitro* and *in vivo* attachment and proliferation of ASCs, BMSC, and chondrocyte have been reported by several studies (Griffon et al., 2006; Elder et al., 2013; Faikrua et al., 2013; Sheehy et al., 2015; Huang et al., 2019; Scalzone et al., 2019). ASCs seeded on a composite hydrogel of silanized-hydroxypropyl methylcellulose (Si-HPMC) mixed with silanized chitosan were found to remain viable both *in vitro* and after subcutaneous implantation in nude mice. Moreover, this ASC-seeded hydrogel exhibited significant regeneration in a canine model of OC defect (Boyer et al., 2020).

In another study, the investigators analyzed ASC-seeded chitosan/gelatin hydrogel and cancellous bone composite scaffold for the regeneration of bone and cartilage. The ASCs were induced to chondrocytes and osteoblasts before implantation in the hydrogel and their subsequent viability, proliferation, and ECM deposition were analyzed. The cells displayed significant proliferation in the composite scaffold and deposited cell-specific ECM which was confirmed by staining and scanning electron microscopy (SEM) (Song et al., 2016). These findings are significant since they can host cells and allow remodeling of its microenvironment, a highly desirable trait for a scaffold.

Articular cartilage relies on its biomechanical and biochemical interplay with the subchondral bone to maintain tissue health (Lories and Luyten, 2011). Therefore, for the regeneration of OC defects, a scaffold that supports the regeneration of cartilage and bone simultaneously is ideal, which is a feature often found missing in general scaffolds. To mitigate this challenge, a poly(l-glutamic acid/chitosan and hydroxyapatite-graft-poly (l-glutamic acid)) scaffold was prepared for the regeneration of both hyaline-like cartilage and underlying subchondral bone. The scaffold was found to support the chondrogenic differentiation of ASCs and spheroid formation. In addition, a chondrogenic ASC spheroid-seeded scaffold successfully regenerated hyaline cartilage along with the underlying subchondral bone in a rabbit-based model of OC defects (Zhang et al., 2016).

### 5.2 Adipose-Derived Stromal/Stem Cell–Calcium Alginate Scaffold

Alginate-based scaffolds with growth factors are widely used for bone (Kolambkar et al., 2011) and cartilage repair (Mierisch et al., 2002). Stem cells are facilitated to differentiate into bone and cartilage cells by using BMP4. It also facilitates the cells to deposit collagen type I and collagen type II and the *in vivo* regeneration of bone and cartilage (Kuroda et al., 2006; Zhang et al., 2008). Calcium alginate (CaAlg) hydrogels fabricated with BMP4-transduced ASCs were found to reconstruct the subchondral bone along with the formation of smooth and flat cartilage surfaces. Because of the upregulation of cytoplasmic BMP4, the secretion of collagen I, collagen II, and alkaline phosphatase was also increased. The deposition of these materials enhanced the differentiation of bone and cartilage cells (Chen et al., 2019).

### 5.3 Adipose-Derived Stromal/Stem Cell–Collagen Scaffold

Type 1 collagen is the most abundant component of the ECM (Yoneno et al., 2005) and a useful material for tissue engineering (Dong and Lv, 2016). A study has reported that the use of scaffolds along with growth factors such as BMP2 enhances the proliferation, attachment, and differentiation of ASCs (Lin et al., 2013). ASCs seeded on a collagen type 1 scaffold have displayed enhanced differentiation to OC lineage compared to two-dimensional (2D) cultures. The differentiation further increased when an ASC-collagen composite was cultured with media supplemented with platelet-rich plasma (PRP) and insulin. The expression of beta-1/beta-3 integrin was increased while it was found that the differentiation was independent of the mammalian target of rapamycin (mTOR) signaling and insulin-like growth factor 1 receptor (IGF-1R) (Scioli et al., 2016).

### 5.4 Adipose-Derived Stromal/Stem Cell–Cartilage-Based Scaffolds

Cartilage-based scaffolds have advantages over other scaffold materials since they contain the native cartilaginous ECM materials crucial for proliferation, attachment, and providing cues for the differentiation of cells. ASCs seeded on cartilage ECM-derived particles (CEDPs) differentiate into chondrocytes without the addition of any exogenous growth factor and with higher efficiency than the 2D-cultured ASCs. ASC-laden CEDPs displayed robust regeneration of rabbit hyaline cartilage, which was limited to fibrous tissue repair only when CEDP was used in the absence of ASCs (Yin et al., 2018).

A biodegradable porous sponge cartilage (BPSC) scaffold has been developed for the regeneration of hyaline-like cartilage. The BPSC scaffold was supplemented with ASCs or its secretome. The BPSC scaffolds fabricated with ASCs were found to regenerate the OC defect more efficiently than the scaffold fabricated with secretome alone (Widhiyanto et al., 2020). Kim et al. (2021) prepared PCL nanofibrils filled with decellularized cartilage ECM which were used along with ASCs. The ASCs seeded on the nanofibrils displayed chondrogenic differentiation without using any exogenous factors and cytokines, which was confirmed by the upregulation of cartilage marker genes. The ASC–nanofibril composite formed a clay-like structure that compactly filled the OC defect in rats and regenerated the cartilage and underlying bone.

Like articular cartilage, auricular cartilage comprises a GAG-collagen type II matrix with limited cell distribution. In contrast to articular cartilage, it has an elastic fiber network which surrounds the cells enabling the uniform distribution of cells. When enzymes are applied to remove the cells, it forms a hollow channel network which enables uniform distribution of cells on repopulation of the decellularized matrix. Bovine auricular cartilage scaffolds were repopulated with bovine and human chondrocytes in monoculture or co-culture with ASCs, which were found to increase cartilage regeneration with a high cell repopulation efficiency (Nürnberger et al., 2019).

### 5.5 Adipose-Derived Stromal/Stem Cell–Oligo (Polyethylene Glycol) Fumarate Scaffold

The oligo (PEG) fumarate (OPF) scaffold has been well characterized in previous studies (Dadsetan et al., 2012). The OPF scaffold has previously shown positive regenerative results in porcine OC defect models, where the scaffold was seeded with BMSCs before implantation (Lim et al., 2013). The OPF scaffold when seeded with autologous and human-derived ASCs exhibited a good quality of regeneration in a pig-based OC defect model compared to an unseeded scaffold. It was also found that type II collagen was expressed at higher levels in the ASCs seeded on an OPF scaffold, while the formation of mature subchondral bone was also observed, characterized by type I collagen expression (De Girolamo et al., 2015).

### 5.6 Adipose-Derived Stromal/Stem Cell–Polycaprolactone Scaffold

Because of its biocompatibility, flexibility, and biodegradability, polycaprolactone (PCL) is one of the most commonly used polyesters in medical applications. PCL-based scaffolds display slow degradation and maintenance of long-term structural integrity during *in vitro* culture. In addition, MSCs derived from the umbilical cord, bone marrow, and adipose tissue differentiate and synthesize bone and cartilaginous matrix when seeded on a PCL scaffold (Kim et al., 2010; Xue et al., 2017). Im and Lee (2010) investigated the comparison of a PCL scaffold seeded with ASCs and immobilized growth factors TGF-β2 and BMP-7 for the regeneration of OC defects in rabbits. Interestingly, the use of growth factors significantly improved the macroscopic scores of the OC defect but failed to improve the histological scores. It was also noted that the ASC-seeded scaffolds had an uncertain effect on the cartilage repair outcome, which was not found to be significantly better than the scaffold alone. The comparable performance of the scaffold alone could have been due to the infiltration of bone marrow stem cells into the scaffold from the surrounding tissue (Im and Lee, 2010).

### 5.7 Adipose-Derived Stromal/Stem Cell-3D Bioprinted Scaffolds

Since it is essential to recapitulate the complex fiber arrangement and pore size for optimal TE, 3D bioprinting has emerged as a useful tool to attain these requirements. A recent study has reported the development of 3D-printed scaffolds that are capable of hosting ASCs and regenerating site-specific OC defects. To drive site-specific ASC osteogenesis and chondrogenesis, a scaffold was fabricated using 3D-bioplotting of biodegradable PCL with TCP, or decellularized bovine cartilage ECM (dECM). The PCL-TCP scaffolds were found to be osteo-inductive whereas the PCL-dECM scaffolds favored chondrogenic differentiation of ASCs. Furthermore, a triphasic full-thickness OC scaffold was developed containing layers of PCL, PCL-TCP, and PCL-dECM to mimic the OC unit. ASCs were seeded on the triphasic scaffold and the histochemical analysis of the scaffold after 28 days of culture revealed that the scaffold was positive for calcium and GAGs in the PCL-TCP and PCL-dECM segments, respectively (Mellor et al., 2020). The *in vivo* evaluation of the same triphasic scaffold in the pig OC defect model revealed that lesions filled with scaffolds along with ASCs showed improved therapeutic effects compared to open lesions. However, although the scaffold facilitated subchondral bone regeneration, cartilage regeneration was found to be limited (Nordberg et al., 2021).

## 6 Adipose-Derived Stromal/Stem Cell–Scaffold Interaction

The interaction of the cells and scaffold is a critical factor in TE that helps the cells grow in natural biomimetic conditions (Chen et al., 2018). For OC regeneration, the scaffold should support the formation of both bone and cartilage throughout the regenerative process and should ideally resorb over time. Several studies have shown that the scaffolds not only allow attachment and proliferation of ASCs but also determine the cellular fate. In addition, the ASCs also remodel their environment while differentiating along different lineages. Scaffold composition, stiffness, porosity, and other physicochemical features have been found to influence the proliferation and differentiation of ASCs. Supplementation of scaffolds with ingredients that support osteogenic or chondrogenic differentiation of ASCs has been found to enhance their OC regenerative potential. A comparison of PLA scaffolds with or without the addition of tricalcium phosphate (TCP) showed that ASCs preferentially underwent osteogenic differentiation in the presence of TCP, whereas higher chondrogenesis was observed in the absence of TCP (Mellor et al., 2015). Biomimetic modifications of the PCL scaffold by the addition of collagen and fibronectin have been found to result in enhanced proliferation, colonization, and osteogenic differentiation of ASCs (Declercq et al., 2014). It has also been observed that ASCs, when seeded on PGA scaffolds, deposit a higher amount of GAGs and total collagen than in pellet culture (Mahmoudifar and Doran, 2010).

The microarchitecture of scaffolds too has the potential to influence cellular activity. ASCs seeded on a PCL scaffold having a modified nanowire surface have been shown to acquire an elongated morphology as opposed to non-elongated morphology when seeded on a smooth surface PCL scaffold. Moreover, ASCs cultured on the nanowire surface PCL scaffold displayed lower chondrogenesis than the smooth PCL scaffold (Trujillo and Popat, 2014). The pore size of the scaffold significantly influences the interaction between cells and the scaffold. The penetration and migration of the cells in scaffolds can be limited when the pore size is too small, whereas if the pore size is too wide, it can hamper cell adhesion (Murphy et al., 2010; Oh et al., 2010). Therefore, optimizing the pore size is critical for the fabrication of an efficient scaffold (Trujillo and Popat, 2014). A study conducted to determine the impact of pore size on ASC function revealed that pores ranging between 370–400 µm in size provide an optimal chondrogenic environment (Gómez et al., 2016).

The remodeling of scaffolds by differentiating ASCs has been reported by several recent studies. Mohiuddin et al. (2019b) showed that osteogenically differentiated ASCs seeded on decellularized adipose matrix remodeled the scaffold by mineral deposition and MMP-mediated rearrangement of ECM fibers. Similarly, other reports suggest that osteogenically and chondrogenically differentiating ASCs deposit calcium phosphates and GAGs, respectively, in the scaffolds (Im et al., 2012; Kim et al., 2021).

## 7 Present Challenges and Future Directions in Adipose-Derived Stromal/Stem Cell-Scaffold-Based Osteochondral Regeneration

### 7.1 Recreating the Bone Cartilage Interface

The calcified layer of cartilage present above the subchondral plate acts as a barrier between the bone and cartilage (Findlay and Kuliwaba, 2016). Some studies have suggested that the communication between the calcified cartilage and subchondral bone occurs through numerous vascular canals (Clark and Huber, 1990). The holes present in the subchondral plate open into the bone marrow space that connects the OC unit (Duncan et al., 1987). Moreover, upon culturing bovine osteochondral explants, it was found that the chondrocytes died more rapidly after 7 days in the absence of subchondral bone, whereas when cultured in the presence of subchondral bone, the chondrocytes remained viable. This phenomenon was potentially a result of the survival factor(s) provided by the bone as observed between the bone and cartilage under physiological conditions (Amin et al., 2009).

Recapitulating the interface between the bone and cartilage is one of the most significant challenges in OC TE. Earlier, some biphasic grafts were developed to support the growth of both bone and cartilage as separate tissues (Chu et al., 1995; Gao et al., 2001; Malda et al., 2005; Getgood et al., 2012). However, they do not optimally mimic the native OC interphase, since they partially support bone and cartilage regeneration in separated scaffold layers (Schek et al., 2004; Keeney and Pandit, 2009). Based on the structure of OC tissue, there is a need for matrices that support tissue regeneration and present an interactive bone–cartilage interface (Athanasiou et al., 2009). To cope with this problem, gradient scaffolds are being developed that support osteogenesis and chondrogenesis, while providing a native tissue-like transition between cartilage and bone layers. These scaffolds have been evaluated both *in vitro* and *in vivo* (Sherwood et al., 2002; Martin et al., 2007; Wang et al., 2009; Mohan et al., 2011; Declercq et al., 2014; Yousefi et al., 2015). A gradient scaffold comprising poly(D, L-lactic-co-glycolic acid), containing TGFβ1 and BMP2, with or without hydroxyapatite, showed a significant extent of OC regeneration in rabbits (Mohan et al., 2011). Several other gradient scaffolds in combination with various cell types have shown promising results for the treatment of OC defects (Du et al., 2017; Gao et al., 2018).

To better understand the intra-articular tissue cross-talk and the etiology of OC defects, Li et al. (2022) have developed an MSC-derived miniature joint system containing OC tissue, fibrous tissue, and adipose tissue. Such systems will not only enhance the understanding of the OC unit but can potentially be adapted to engineer patient-specific OC units for transplantation in the future.

### 7.2 Availability of Clinical-Grade Adipose-Derived Stromal/Stem Cells

ASCs are a good candidate for OC defect regeneration because of their easy availability, rich source, and biocompatibility with various types of scaffolds. However, some limitations must be addressed for the successful clinical translation of ASC-scaffold composites. The use of autologous ASCs is considered safe for therapeutic applications as they are not expected to elicit an immune response. However, obtaining a sufficient amount of tissue to isolate a clinically efficacious number of cells is a major challenge (Zhang et al., 2015). To overcome this issue, ASCs can be culture-expanded to increase the cell number (Kharbanda et al., 2014). Another potential solution is the use of allogeneic stem cells. However, the availability of adipose tissue is dependent on surgical procedures, which limits the availability of tissue from healthy donors. Another limitation in using allogeneic ASCs is the variability at the cellular and molecular levels as a consequence of donor sex, age, BMI, and tissue depot (Shu et al., 2012; Bodle et al., 2014; Ock et al., 2016; Abbo et al., 2017).

To produce clinical-grade ASCs, good manufacturing practice (GMP)-based manufacturing facilities need to be established where ASCs obtained from healthy donors are isolated and culture-expanded under aseptic conditions. The ASCs isolated from multiple donors can then be pooled to minimize donor-based variability in the final batch of cells to be used for therapeutic purposes (Kuçi et al., 2016) (Ankrum et al., 2014; Patrikoski et al., 2014). A major impediment in the large-scale culture of clinical-grade cells is the availability of a non-xenogeneic source of growth factors. Animal-derived serum is presently the gold standard for experimental cell culture; however, for clinical translation, human serum and platelet lysate are being analyzed.

The optimal concentration of ASCs to be seeded on scaffolds for OC regeneration is another area that needs increased focus and harmonization. A wide range of ASC doses has been tested for OC regeneration thus far, details of which are shown in Table 1. For the clinical translation of ASC-scaffold composites, it is imperative that future studies are conducted to determine the dose-specific effect of these composites on OC regeneration.

**TABLE 1:** *In vitro* and preclinical application of ASC-scaffold composites.

S. No.	ASC dose	Source of ASCs	Scaffold type	Model	Reference
1	2 × 10^6^/ml	Human	Silylated chitosan and cellulose hydrogel	Canine OC defect in dogs	Boyer et al. (2020)
2	0.02 × 10^6^ cells/spheroid	Human	3D-printed osteochondral interface using osteogenic and chondrogenic spheroid	In vitro study	Ayan et al. (2020)
3	0.5 × 10^6^ cell/40 ml	Rabbit	Porous poly (ε-caprolactone) (PCL) scaffold with different pore sizes	OC defect in femur of rabbit	Im et al. (2012)
4	0.2 × 10^6^ cells/350 μl	Human	3D collagen gel scaffold	In vitro study	Scioli et al. (2016)
5	1 × 10^7^ cells/ml	Rabbit	Coacervate-embedded composite hydrogels	Femoral trochlear osteochondral defect in rabbit	Cho et al. (2020)
6	0.25 × 10^6^/cm^2^ (when 0% tricalcium phosphate used) and 0.02 × 10^6^/cm^2^ (when 20% TCP used)	Human	Stacked polylactic acid nanofibrous scaffolds	In vitro study	Mellor et al. (2015)
7	1 × 10^7^ cells/ml	Human	Cancellous bone/hydrogel (chitosan/gelatin) hybrid scaffold	In vitro study	Song et al. (2016)
8	2 × 10^6^ cells	Rabbit	Cartilage extracellular matrix-derived particles (CEDPs) and cartilage slice-based scaffold	Rabbit femoral trochlear osteochondral defect	Yin et al. (2018)
9	0.4 × 10^6^ cells/30 ul	Human immortalized ASCs (Evercyte, Cat# CHT-001-0005)	Photo-crosslinked gelatin methacryloyl (gelMA) scaffold	In vitro study	Hölzl et al. (2021)
10	0.256 × 10^6^ per microspheroids	Human immortalized ASCs (Evercyte, Cat# CHT-001-0005)	Gelatin-based hydrogels	In vitro study	Zigon-Branc et al. (2019)
11	0.25 × 10^6^ cells for the chondrogenic layer and 0.5 × 10^6^ cells for the osteogenic layer	Rabbit	Trilayered silk fibroin scaffolds	In vitro study	Ding et al. (2014)
12	0.25 × 10^6^ cells/scaffold	Human	Multiphasic 3D-bioplotted scaffolds	In vitro study	Mellor et al. (2020)
13	2 × 10^6^ cells/scaffold	Rabbit	Biodegradable porous sponge cartilage scaffold	Full thickness femoral defect in rabbits	Widhiyanto et al. (2020)
14	0.5 × 10^6^ cells/ml (in vivo) 5 × 10^3^ cells/ml–0.5 × 10^6^ cells/ml (in vitro)	Not specified	Protein-reactive nanofibrils scaffold	Articular cartilage defect	Kim et al. (2021)
15	50 ul of 50 × 10^6^ cells/ml	Rabbit	Poly(L-glutamic acid)-based scaffold	Articular osteochondral defect in rabbits	Zhang et al. (2016)
16	3 × 10^6^ cells	Minipig human	Oligo (polyethylene glycol) fumarate (OPF) hydrogel	OC defect in minipigs	De Girolamo et al. (2015)
17	0.5 × 10^6^ cells	Rabbit	Immobilized porous polycaprolactone scaffold	Distal femur OC defect in rabbits	Im and Lee, (2010)
18	0.25 × 10^6^ co-cultured cells with human articular chondrocytes	Human immortalized ASCs (Evercyte, Cat# CHT-001-0005)	AuriScaff (auricular cartilage scaffold)	OC plug model in mice	Nürnberger et al. (2019)
19	0.075 × 10^6^ and 0.15 × 10^6^ cells	Pig ASCs	Multiphasic 3D-bioplotted scaffold	OC defects in minipigs	Nordberg et al. (2021)

### 7.3 Elucidation of Adipose-Derived Stromal/Stem Cell Paracrine Activity

Immunomodulatory and anti-inflammatory capacities are the most clinically relevant properties of ASCs. Several studies have confirmed that ASCs carry out these activities via paracrine signaling via the upregulation of anti-inflammatory cytokines while suppressing pro-inflammatory molecules (Melief et al., 2013; Carrade Holt et al., 2014; Bowles et al., 2017). ASCs have been found to suppress the proliferation and migration of activated inflammatory cells in case of arthritis, thus preventing bone and cartilage degradation (Ter Huurne et al., 2012; Manferdini et al., 2017; Ueyama et al., 2020). Although the role of ASC paracrine activity in tissue generation *via* modulation of immune and inflammatory response is well established, the present literature relating to the use of ASC-scaffold composites for OC regeneration has placed very little emphasis on the elucidation of these mechanisms. Further studies are mandatory to establish the understanding of molecular mechanisms involved in the regeneration of OC defects by these composites. Moreover, it will be interesting to evaluate if ASCs display enhanced or diminished paracrine activity when implanted alone or in combination with scaffolds.

## 8 Clinical Trials

Based on our search using www.clinicaltrials.gov, twelve clinical trials have been initiated to test the OC regenerative capability of the scaffolds. These studies involve the use of scaffolds and/or different types of cells. Details of the clinical trials are provided in Table 2 (www.clinicaltrials.gov).

**TABLE 2:** Clinical trials for osteochondral defects.

S. No.	Title	Condition	Scaffold used	No. of participants	Study start date	Expected end date	Country	Status
1	Repair of Articular Osteochondral Defect	Osteochondritis dissecans	Biphasic osteochondral composite	10	March 2009	December 2011	Taiwan	Unknown status
2	A Study to Evaluate the Efficacy of BioCartilage® Micronized Cartilage Matrix in Microfracture Treatment of Osteochondral Defects	Osteochondral defect	BioCartilage® micronized cartilage matrix	15	January 2019	November 2023	Canada	Recruiting
3	Follow-up Study Evaluating the Long-Term Outcome of ChondroMimetic in the Treatment of Osteochondral Defects in the Knee	Osteochondral defect	ChondroMimetic	15	May 2017	February 2018	Hungary	Completed
4	A Prospective, Post-Marketing Registry on the Use of ChondroMimetic for the Repair of Osteochondral Defects	Osteochondral defects	Chondromimetic	8	September 2010	April 2013	Hungary	Terminated
5	A Study to Evaluate the Safety of Augment™ Bone Graft	Defect of articular cartilage	Augment Bone Graft	1	July 2011	August 2012	Canada	Completed
6	Transplantation of Bone Marrow Stem Cells Stimulated by Protein Scaffolds to Heal Defects in Articular Cartilage of the Knee	Osteoarthritis|knee Osteoarthritis|osteochondritis	Transplantation of bone marrow stem cells	50	July 2010	December 2014	France	Unknown status
7	The Effectiveness of Adding Allogenic Stem Cells After Traditional Treatment of Osteochondral Lesions of the Talus (OLT)	Osteochondral fracture of talus	Allogenic stromal mesenchymal cells derived from the umbilical cord | platelet-poor plasma Scaffold	70	15 January 2019	December 2024	Chile	Recruiting
8	One-Step Bone Marrow Mononuclear Cell Transplantation in Talar Osteochondral lesions	Osteochondritis	Procedure: bone marrow cells transplantation on collagen scaffold	140	April 2013	April 2018	Italy	Unknown
9	Study for the Treatment of Knee Chondral and Osteochondral Lesions	Knee chondral lesion knee osteochondral lesion	Procedure: Marrow stimulation—drilling or microfractures device: MaioRegen surgery	145	January 2011	February 2016	Europe and South Africa	Completed
10	Triphasic Osteochondral Scaffold for the Treatment of the OCD of the Knee: Observational Study	Osteochondritis dissecans knee	Triphasic Scaffold	30	1 April 2022	April 2029	Italy	Recruiting
11	Evaluation of an Acellular Osteochondral Graft for Cartilage Lesions Pilot Trial (EAGLE Pilot)	Articular cartilage injury	Device: Kensey Nash Corp. Cartilage repair device	2	June 2010	May 2014	United States	Terminated
12	Biphasic Cartilage Repair Implant (BiCRI) IDE Clinical Trial-Taiwan	Chondral or osteochondral lesion of medial femoral condyle chondral or osteochondral lesion of lateral femoral condyle chondral or osteochondral lesion of trochlea	Device: Biphasic cartilage repair implant	92	October 2011	August 2019	Taiwan	Completed

## 9 Conclusion

Despite the recent advancements in OC tissue engineering, there is still a need to optimize natural and synthetic biomaterials that can repair OC defects. The scaffolds when implanted along with the ASCs show a higher regenerative efficacy than the use of ASCs or scaffolds alone. However, presently, there is an advantage of using scaffolds alone as a cell-free system as it mitigates the regulatory complications related to the application of ASCs. Most of the current studies to treat OC defects are in the experimental phase. There is a need to carry out clinical trials translating promising results from pre-clinical animal models. To date, few clinical trials have been conducted thus far to analyze the safety and efficacy of ASC-scaffold composites. With the completion of present ongoing clinical trials and others to follow, there will be greater clarity about the future course of action in terms of the optimal scaffold design and cell seeding strategy. An ideal OC scaffold should support both osteogenesis and chondrogenesis, which while being interrelated, are distinct phenomena. A promising future strategy appears to be the development of layered scaffolds which contain osteogenic and chondrogenic compartments as reported by Mellor et al. (2020) and Nordberg et al. (2021). Such scaffolds recapitulate the physiological OC structure and are therefore exciting prospects for the regeneration of OC defects and *in vitro* OC engineering. Moreover, the availability of clinical-grade allogeneic ASCs is also required to advance the field, since autologous ASCs are generally not available in sufficient numbers and quality to efficiently regenerate physiological defects.

Overall, while the development of ASC-scaffold composites for OC regeneration is in its early stages of research, the available results are promising but will require further validation in human patients under protocols approved and evaluated by internationally recognized regulatory authorities.
